# Tissue Distribution and Abundance of the Parasitic Dinoflagellate *Hematodinium perezi* in Naturally Infected *Portunus trituberculatus*

**DOI:** 10.3390/pathogens14070650

**Published:** 2025-06-30

**Authors:** Ju Zhang, Meng Li, Qian Huang, Lijun Hu, Qi Xue, Jiayi Wang, Caiwen Li

**Affiliations:** 1 CAS Key Laboratory of Marine Ecology and Environmental Sciences, Institute of Oceanology, Chinese Academy of Sciences, Qingdao 266071, China; zhangju@qdio.ac.cn (J.Z.); huangqian@qdio.ac.cn (Q.H.); hulijun@qdio.ac.cn (L.H.); xueqi@qdio.ac.cn (Q.X.); wangjiayi@qdio.ac.cn (J.W.); 2 University of Chinese Academy of Sciences, Beijing 100049, China; 3 Laboratory for Marine Ecology and Environmental Science, Qingdao Marine Science and Technology Center, Qingdao 266237, China; 4 Centre for Ocean Mega-Science, Chinese Academy of Sciences, Qingdao 266071, China

**Keywords:** parasitic dinoflagellate, *Hematodinium perezi*, life stages, abundance, tissue distribution, *Portunus trituberculatus*

## Abstract

The parasitic dinoflagellate *Hematodinium* is an infectious pathogen that causes severe enzootic in numerous economically important marine crustaceans worldwide. Previous research has focused on investigating the identification and life stages of *Hematodinium* parasites, while the parasite abundance and tissue proliferation process of *Hematodinium* in naturally infected crustacean hosts need to be further studied. In the present study, the tissue tropisms and intensity of *H. perezi* were investigated in the naturally infected Chinese swimming crabs *Portunus trituberculatus* by both the qualitative (hemolymph assay, histology) and quantitative analysis (cell count, quantitative PCR). The results showed that in *P. trituberculatus* with infection level I (4 ± 2 parasites in 200× microscopic field), filamentous trophonts were observed in the hemolymph and stomach tissues, with the average parasite number and ITS 1 copy number of *H. perezi* quantitatively detected in hemolymph (1.0 × 10^2^ parasites/mL) and stomach tissues (1.7 × 10^3^ cells/g), respectively. *H. perezi* trophonts were observed in the hemolymph (4.3 × 10^4^ parasites/mL) and exhibited broad distribution in multiple tissues with its highest abundance of *H. perezi* in pereiopod muscles (1.1 × 10^4^ cells/g) followed by that in stomach (4.8 × 10^3^ cells/g) in *P. trituberculatus* with infection level II (80 ± 10 parasites in 200× microscopic field). In *P. trituberculatus* with infection level III (200 ± 35 parasites in 200× microscopic field), a high abundance of *H. perezi* sporoblasts was found in the hemolymph (3.1 × 10^7^ parasites/mL) and all of the other examined tissues, with its highest abundance detected in pereiopod muscles (3.5 × 10^4^ cells/g). In addition, the number of host’s hemocytes was significantly decreased during the *Hematodinium* infection. This study provides a comprehensive quantitative characterization of the tissue distribution and abundance of *H. perezi* in its natural crab host which will contribute to better understanding of the crustacean host–*Hematodinium* interactions.

## 1. Introduction

The parasitic dinoflagellate *Hematodinium* is a pathogenic parasite affecting a wide range of marine crustaceans globally [1,2,3]. To date, there are only two formally described species in the genus *Hematodinium*, including the type species, *Hematodinium perezi* [4], and the second species, *Hematodinium australis* [5]. Since *Hematodinium* was originally reported in *Carcinus maenas* and *Liocarcinus depurator* [4], *Hematodinium* or *Hematodinium*-like parasites have been found and reported to infect over 70 crustacean species [6]. *Hematodinium* primarily parasitizes and proliferates in the hemolymph or hemocoel of its crustacean hosts, resulting in the dysfunction of major organs (e.g., heart, hepatopancreas, and gills) and ultimately leading to host mortality [7,8,9]. In past decades, *Hematodinium* enzootic has frequently occurred and caused large economic loss in many commercially important wild and cultured crustaceans, such as the blue crab, *Callinectes sapidus* [7,10], the Norway lobster, *Nephrops norvegicus* [11,12,13], shore crabs, *Carcinus maenas* [14], edible crabs, *Cancer pagurus* [15,16], the tanner crab, *Chionoecetes bairdi* [17,18], the mud crab, *Scylla paramamosain* [19], and swimming crabs, *Portunus trituberculatus* [20,21,22].

Shandong Peninsula is one of the major culture regions of the Chinese swimming crab *P. trituberculatus*, where more than 19,255 t were harvested in 2023, accounting for 20% of the total production in China [23]. In the mariculture areas in Huangdao (Qingdao, China), *Hematodinium* infections were observed in juvenile *P. trituberculatus* after they were added to the polyculture ponds, and infections were persistent in the polyculture pond from June until October, with peak prevalence (up to 90%) observed in late July to early August, and most of them were moderately or heavily infected individuals [20,24]. Outbreaks of *Hematodinium* enzootic have caused substantial economic losses to local aquaculture farmers.

The life cycle of *Hematodinium* is complex and has been explored by in vitro or in vivo studies since its first presence reported by Chatton and Possion [4]. Then, Appleton and Vickerman [25] described the complete life cycle of *Hematodinium* sp. isolated from *N. norvegicus.* Li et al. depicted the life history of *H. perezi* from *C. sapidus* and identified the life stage of schizonts [26]. Gaudet et al. achieved the in vitro culturing of *Hematodinium* sp. isolated from *C. opilio* and characterized its life stages including trophonts, clump colonies, sporonts, arachnoid sporonts, sporoblasts, and dinospores [27]. In addition, the life stages of *H. perezi* isolated from *P. trituberculatus* and *H. tientsinensis* were identified and depicted by in vivo and in vitro experiments [20,24,28]. *Hematodinium* isolated from various hosts were observed to generally experience several life stages including filamentous trophonts, amoeboid trophonts, arachnoid trophonts, sporonts, sporoblasts, prespores, and dinospores [20,24,25,26] (Supplementary Table S1). The dinospores are considered the initial stage of the *Hematodinium* life cycle, developing first to filamentous trophonts [20,25,26]. Amoeboid trophonts subsequently arise from merogony of the filamentous trophonts, further developing into arachnoid trophonts and arachnoid sporonts. Sporoblasts are released from fully developed arachnoid sporonts. As the life stages progress, motile macro/micro-dinospores develop from the prespores [25,26].

In addition, *Hematodinium* undergoes several in vivo life stages in the infected *Chionoecetes bairdi* hemolymph including trophonts, prespores, and dinospores [29]. Uninucleate and plasmodial trophonts of *Hematodinium* parasites were observed in the hemolymph and connective tissues of infected *C. maenas* [14]. Uni- and multi-nucleate trophonts in the hemolymph of juvenile *Cancer pagurus* artificially inoculated with *Hematodinium* parasites were also observed [16]. Wang et al. focused on identifying the representative life stages of *H. perezi* in hemolymph and heart tissues and investigating the histopathological alteration in four tissues (hepatopancreas, heart, gills, and muscle) of *H. perezi*-infected *P. trituberculatus* by histology analysis [28]. Lyu et al. studied the representative life stages of *H. perezi* in the hemolymph, hepatopancreas, heart, and gills of *H. perezi*-infected *Helice tientsinensis* by histology analysis [24]. Although previous studies have been focused on qualitative analysis of the *Hematodinium* life stages in crustacean hosts via in vivo and in vitro experiments, the parasite abundance and tissue proliferation of *Hematodinium* parasites inside naturally *H. perezi*-infected crustacean hosts has scarcely been studied.

Thus, the present study aimed to characterize the abundance and tissue distribution of *Hematodinium* parasites in as many as eight tissues of naturally *H. perezi*-infected *P. trituberculatus*. We distinguished the parasite abundance by cell counting and qPCR and evaluated the extent of tissue involvement through histological analysis. This combined approach not only provided a more comprehensive understanding of the parasite’s life cycle and tissue distribution but also offered quantitative insights into the progression of infection. This research provides a reference for exploring the infection process of *H. perezi* and the interaction between *H. perezi* and its crustacean host.

## 2. Materials and Methods

### 2.1. Collection of Experimental Crabs and Molecular Identification of Hematodinium Species

A total of 60 male *Portunus trituberculatus* (wet weight: 60 ± 10 g; carapace width: 99.3 ± 6.7 mm) were purchased from an aquaculture farm (Jiaonan, Shandong Province, China) from June to July 2023. The crabs were transported to the laboratory at the Institute of Oceanology, Chinese Academy of Sciences (IOCAS), in aerated moisture chambers for the subsequent diagnosis of *H. perezi* infections. Before the laboratory treatment, the crabs were temporarily housed in the blue plastic tanks equipped with a circulating filtered seawater system (salinity of 30, temperature of 23 °C) with oxygen supply and fed with fresh clams twice a week. To maintain optimal water quality, approximately 30% of the seawater in the circulating system was replaced twice a week. To prevent the potential cross-infection, the infected and healthy crabs were housed separately in the circulating seawater system during the experiment. Infection status was determined prior to grouping by examining hemolymph samples of *P. trituberculatus* using light microscopy to detect the presence of *Hematodinium* parasites.

The *Hematodinium* species infecting *P. trituberculatus* in this study was identified as *H. perezi* by using the PCR assay as described in [21]. Briefly, genomic DNA was extracted from hemolymph samples of *Hematodinium*-infected *P. trituberculatus* (*n* = 6) using a TIANamp Marine Animals DNA Kit (Tiangen, Beijing, China). The first internal transcribed spacer (ITS1) of the *Hematodinium* ribosomal DNA was amplified with a specific primer set (forward primer: 5′-CATTCACCGTGAACCTTAGCC-3′; reverse primer: 5′-CTAGTCATACGTTTGAAGAAAGCC-3′) [30]. The PCR amplification reactions were performed as follows: initial denaturation at 94 °C for 5 min, followed by 35 cycles of 30 s at 94 °C, 30 s at 56 °C, and 1 min at 72 °C, with a final extension at 72 °C for 5 min. The PCR products of ITS 1 rDNA were sequenced by Sangon Biotech (Shanghai, China). The sequences were subjected to the Nucleotide BLAST in the NCBI database.

### 2.2. Microscopic Diagnosis of H. perezi Infection

The Chinese swimming crabs were examined to detect *H. perezi* infection using the hemolymph assay as described in a previous study [20]. Briefly, 2–3 drops of hemolymph extracted from the fifth leg of the swimming crabs were mixed with an equal volume of 0.04% neutral red in filtered seawater on a glass slide. The glass slide was then screened by using a light microscope (Olympus BX53, Tokyo, Japan) at 200× and 400× magnification for *H. perezi* presence. According to the previous study [20], the average parasite number (mean ± SD) in the field of the microscopic view under 200× magnification was counted from three randomly selected fields per slide. One slide was prepared and examined for each crab. Based on these counts, infection levels were classified into three categories, including infection levels I (4 ± 2 parasites), II (80 ± 10 parasites), and III (200 ± 35 parasites) (Supplementary Table S2). Hemolymph and eight different tissues (epidermis, hepatopancreas, gills, cheliped muscle, pereiopod muscle, heart, stomach, and eyestalks) were collected from crabs with the three different natural *H. perezi* infection levels (*n* = 6 for each infection level). After being thoroughly washed three times with 1 × PBS buffer solution, the tissue samples were divided into halves, with one half stored immediately at −80 °C after freezing in liquid nitrogen for subsequent genomic DNA extraction, while the other half was fixed in Bouin’s fixative solution (prepared with filtered seawater) for histopathological analysis.

### 2.3. H. perezi and Hemocyte Cell Counts in Hemolymph of P. trituberculatus with Different Infection Levels

The hemolymph sample (~100 μL) from each naturally *H. perezi*-infected crab was collected and mixed homogeneously with an equal volume of 0.04% neutral red solution. Subsequently, ~20 μL of the mixed solution was added to a hemocytometer (Hausser Scientific, Horsham, PA, USA) to count the number of *H. perezi* parasites and host hemocytes by using a light microscope at 200× magnification (Olympus BX53, Tokyo, Japan). Each hemolymph sample was counted in triplicate. In addition, the life stages of *H. perezi* present in the hemolymph of *P. trituberculatus* were recorded carefully by the hemolymph assay as described above.

### 2.4. Histopathological Diagnosis of H. perezi Infections in Different P. trituberculatus Tissues

The eight tissues sampled from naturally *H. perezi*-infected *P. trituberculatus* with three different infection levels (I–III) as mentioned in Section 2.2 was fixed and subjected to H&E staining histological analysis as described in [9]. After 48 h, the fixed samples were rinsed thoroughly by immersion in 70% ethanol. The ethanol solution was replaced every 2 h over a period of 12–24 h until the yellow coloration from picric acid was no longer visible. Subsequently, gradient dehydration was carried out in the order of 70% ethanol for 1 h, 80% ethanol for 1 h, 90% ethanol for 1 h, and anhydrous ethanol for 40 min. The samples were then embedded with paraffin wax and stained with Mayer’s hematoxylin and eosin (H&E), following the protocol detailed in [9]. The prepared slides were sealed with neutral resin, photographed, and observed with the microscope at 100× or 200× (Olympus BX53, Tokyo, Japan) equipped with an Olympus DP73 digital camera.

### 2.5. Quantitative Analysis of H. perezi Infection in Different P. trituberculatus Tissues

Quantitative PCR (qPCR) was employed to assess the abundance of *H. perezi* in various host’s tissues. Genomic DNA was extracted from an equal 100 mg of *H. perezi*-infected tissues by using a TIANamp Marine Animals DNA Kit (TIANGEN, Beijing, China) according to the manufacturer’s instructions. The qPCR was carried out with a ChamQ Universal SYBR qPCR Master Mix kit (Vazyme, Nanjing, China) using the Rotor-Gene Q 2plex HRM thermocycler (QIAGEN, Düsseldorf, Germany). The specific primers ITS1-F (5′-CATTCACCGTGAACCTTAGCC-3′) and ITS1-R (5′-CTAGTCATACGTTTGAAGAAAGCC-3′) were used to amplify the first internal transcribed spacer (ITS1) of *H. perezi* ribosomal DNA [30]. The PCR amplification reaction was performed as follows: initial denaturation at 94 °C for 5 min, followed by 35 cycles of 30 s at 94 °C, 30 s at 56 °C, and 1 min at 72 °C, with a final extension at 72 °C for 5 min.

To establish a standard curve for the ITS1 of *H. perezi*, the *H. perezi* ITS1 rDNA sequence was amplified using the genomic DNA extracted from the heavily infected crab hemolymph. A standard curve was generated using a serial dilution of plasmid DNA containing ITS1 amplicons (10^9^ to 10^2^ copies per reaction) of *H. perezi* according to [31]. The standard curve of *H. perezi* ITS1 copies was established using the C_T_ values and log copy number of *H. perezi* ITS1, and the regression equation was obtained as Y = −0.3386X + 11.314 (R^2^ = 0.9877) (Figure S1). Then, *H. perezi* ITS 1 in the tissues of *H. perezi*—infected hosts was quantified by qPCR analysis, and C_T_ values were converted to ITS 1 copy numbers by using the standard curve regression equation. Finally, the *H. perezi* ITS1 copy number in the sampled tissues with equal weight was converted into parasite intensity (parasites per gram of tissue) with ~300 ITS1 copies representing one parasite cell as indicated in [31].

### 2.6. Statistical Analysis

All data were displayed as the mean ± standard deviation (mean ± SD). Prior to statistical analysis, data normality was assessed using the Shapiro–Wilk test, and the homogeneity of variances was evaluated using Levene’s test [32]. For comparisons among groups, one-way analysis of variance (ANOVA) was performed using GraphPad Prism 5 (GraphPad Software, San Diego, CA, USA). When ANOVA assumptions were not met, non-parametric alternatives were applied. Statistical significance was defined as *p* < 0.05.

## 3. Results

### 3.1. Life Stages and Cell-Count-Based Quantification of H. perezi in Hemolymph of P. trituberculatus with Different Infection Levels

**Figure 1:**
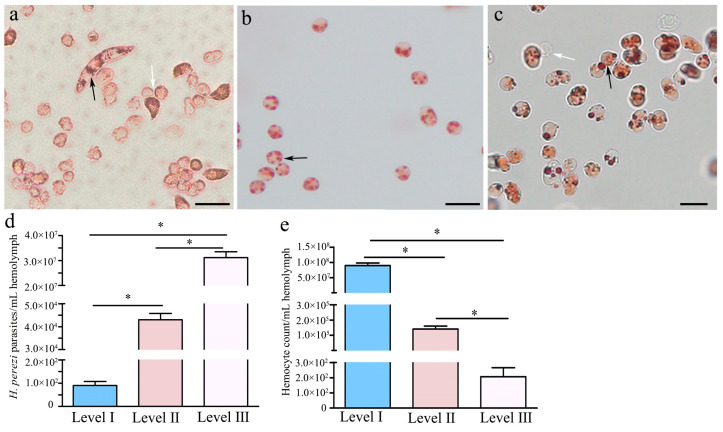
**The life stages and abundance of *H. perezi* in the hemolymph of *Portunus trituberculatus* with different infection levels.** (**a**) Filamentous trophonts in the hemolymph of *Portunus trituberculatus* with infection level I. (**b**) Round trophonts in the hemolymph of individuals with infection level II. (**c**) Sporoblasts in the hemolymph of individuals with infection level III. *H. perezi* cells and hemocytes are indicated by black and white arrows, respectively. *H. perezi* density (**d**) and total hemocyte counts (**e**) in the hemolymph of *Portunus trituberculatus* with different infection levels. Data are presented as the mean ± SD (*n* = 6). Significant differences (*p* < 0.05) are indicated by the asterisks. Scale bar = 20 μm.

By qualitative analysis with hemolymph assays, the filamentous trophonts, round trophonts, and sporoblasts of *H. perezi* were, respectively, identified in the hemolymph of *P. trituberculatus* with infection levels I (Figure 1a), II (Figure 1b), and III (Figure 1c). The average parasite numbers in *H. perezi* were counted by using a hemocytometer (Hausser Scientific, Horsham, PA, USA) (Figure 1d) and found to be 100 parasites/mL in the hemolymph of *P. trituberculatus* with infection level I, 4.3 × 10^4^ parasites/mL in the hemolymph of individuals with infection level II, and 3.1 × 10^7^ parasites/mL in the hemolymph of individuals with infection level III. In addition, the cell intensities of *P. trituberculatus* hemocytes were also counted in this study and found to be 9.0 × 10^7^, 1.4 × 10^5^, and 2.1 × 10^2^ cells/mL in *P. trituberculatus* with infection levels I, II, and III, respectively (Figure 1e).

### 3.2. Tissue Distribution of H. perezi in P. trituberculatus with Different Infection Levels

#### 3.2.1. Tissue Distribution of *H. perezi* in *P. trituberculatus* with Infection Level I

**Figure 2:**
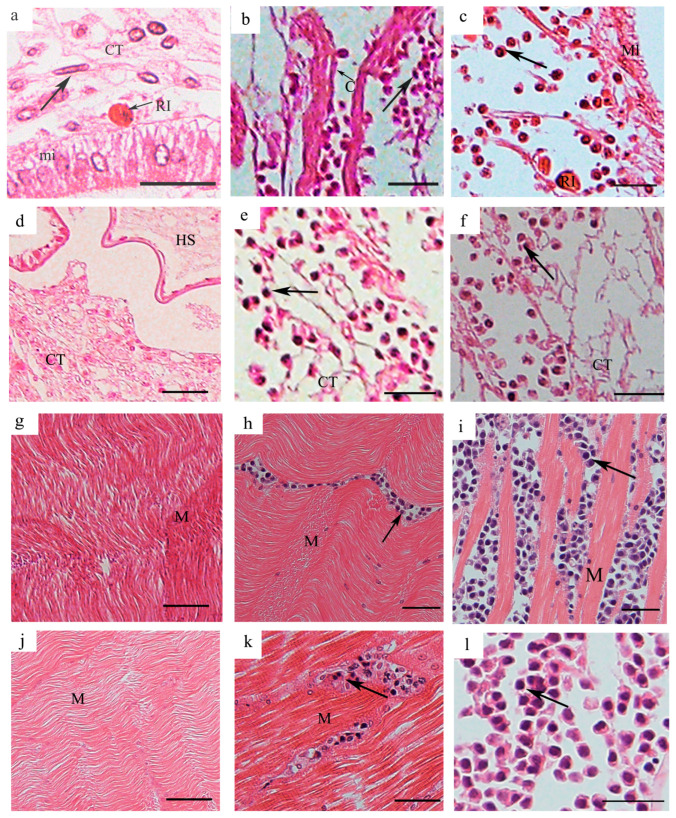
**Histopathology of the stomach, epidermis, cheliped muscle, and pereiopod muscle in *P. trituberculatus* with different infection levels.** The histopathology of the stomach in individuals with infection levels I (**a**), II (**b**), and III (**c**). The histopathology of the epidermis in individuals with infection levels I (**d**), II (**e**), and III (**f**). The histopathology of the cheliped muscle in individuals with infection levels I (**g**), II (**h**), and III (**i**). The histopathology of the pereiopod muscle in individuals with infection levels I (**j**), II (**k**), and III (**l**). *H. perezi* cells are indicated by the black arrows. CT, connective tissue; mi, midgut epithelium; Ml, membranous layer; M, muscle; CT, connective tissue; HS, hemal space. Scale bar = 50 μm.

**Figure 3:**
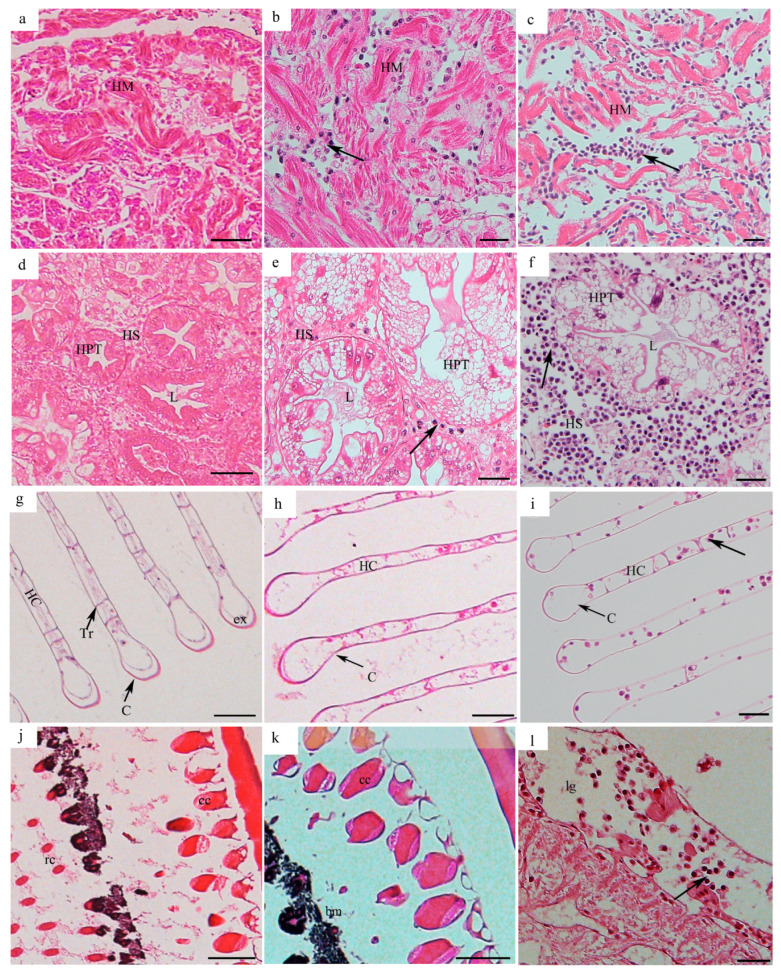
**The histopathology of the heart, hepatopancreas, gills, and eyestalk in *P. trituberculatus* with different infection levels.** The histopathology of the heart in individuals with infection levels I (**a**), II (**b**), and III (**c**). The histopathology of the hepatopancreas in individuals with infection levels I (**d**), II (**e**), and III (**f**). The histopathology of the gills in individuals with infection levels I (**g**), II (**h**), and III (**i**). The histopathology of the eyestalks in individuals with infection levels I (**j**), II (**k**), and III (**l**). *H. perezi* cells are indicated by the black arrows. C, cuticular tissue; HPT, hepatopancreatic tubules; L, lumen; HC, hemal channel; Tr, trabecular cells; ex, exocuticle; rc, retinular cells and rhabdom; cc, crystalline cone; bm, basement membrane; lg, lamina ganglionaris. Scale bar = 50 μm.

The histopathological diagnosis of different tissues in *P. trituberculatus* with infection level I showed no significant structural alterations in all of the examined tissues. At *H. perezi* infection level I, midgut epithelia and underlying connective tissue within the stomach were clearly observed and well-structured with only filamentous trophonts found in the connective tissues (Figure 2a). The underlying connective tissue of epidermis showed no significant pathological alteration with hemal spaces well-defined and sporadic hemocytes observed (Figure 2d). In addition, the muscle bundled and was closely associated with the epithelial layer (Figure 2d). The muscle fibers in the cheliped and pereiopod muscles were arranged tightly with distinct banded structures (Figure 2g,j). Myocardial fibers were tightly arranged with well-defined hemal spaces (HS) (Figure 3a). Intact hepatopancreatic tubules were observed and interconnected by loose connective tissues and hemal sinus, and the lumens of hepatopancreatic tubules were narrow with the tubules packed densely (Figure 3d). The gill lamellae exhibited intact structures with the hemal channels formed by trabecular cells and sporadic hemocytes in the lamellae; the structures of the cuticle and exocuticle remained intact and no *H. perezi* cells were found in the gill hemal channel (Figure 3g). The eyestalk exhibited structural integrity in the cuticle and basement membrane, with intact crystalline cone cells, and the retinular cells and rhabdoms (rc) were arranged in an orderly manner (Figure 3j).

#### 3.2.2. Tissue Distribution of *H. perezi* in *P. trituberculatus* with Infection Level II

*H. perezi* trophonts were clearly observed in the stomach, epidermis, heart, hepatopancreas, cheliped, and pereiopod muscles of *P. trituberculatus* with infection level II and caused significant histopathological alterations. The underlying connective tissue in the stomach was disrupted due to the infiltration of *H. perezi* trophonts, while the cuticle was structurally intact and clearly found (Figure 2b). The *H. perezi* trophonts seemed to make the connective tissues of the epidermis fragmented and disorganized (Figure 2e). Localized muscle fiber breakage and separation was found in the muscles with *H. perezi* trophonts filled in the muscle fiber spaces (Figure 2h,k). *H. perezi* trophonts infiltrated the interstitial spaces of the myocardial fibers and widened the myofilament spaces and partial lytic degeneration of the fibers (Figure 3b). The hepatopancreatic tubules were enlarged by the infiltration of *H. perezi* trophonts with the connective tissues disrupted (Figure 3e). The gill filament tips were swollen, gill hemal channels become distorted, and trabecular were damaged, although *H. perezi* cells were still not visible (Figure 3h). The eyestalk exhibited structural integrity in the cuticle and basement membrane, and no *H. perezi* cells were observed in the eyestalk (Figure 3k).

#### 3.2.3. Tissue Distribution of *H. perezi* in *P. trituberculatus* with Infection Level III

The *H. perezi* parasites were distributed widely in all of the examined tissues of *P. trituberculatus* with infection level III and caused significant histopathological alterations in the hosts. The connective tissues of the stomach were extensively disrupted and even lost, and the hemal sinus was infiltrated with abundant *H. perezi* and few hemocytes (Figure 2c). The parasite was observed in the hemal space of the epidermis with the hemocytes significantly diminished (Figure 2f). The cheliped and pereiopod muscles exhibited extensive degeneration and lost their normal tightly packed banding pattern with muscle fibers significantly separated, fragmented, and dissolved (Figure 2i,l). The myocardium of the heart was filled with a large number of *H. perezi* cells, and the myocardial fibers were loose and separated from each other (Figure 3c). The hepatopancreatic tissue was extensively infiltrated with abundant *H. perezi*, which resulted in the severe dilation of hepatopancreatic tubules and loss of connective tissues (Figure 3f). Gill filaments were infiltrated with *H. perezi*, which resulted in a thinner and necrotic epithelium. The lamellas at the ends of the gills were swelled significantly and the trabecular cells were destroyed due to the infiltration of *H. perezi* (Figure 3i) and *H. perezi* parasites were also observed in the lamina ganglionaris of the eyestalks (Figure 3l).

### 3.3. qPCR Assay of H. perezi Abundance in P. trituberculatus Tissues with Different Infection Levels

**Figure 4:**
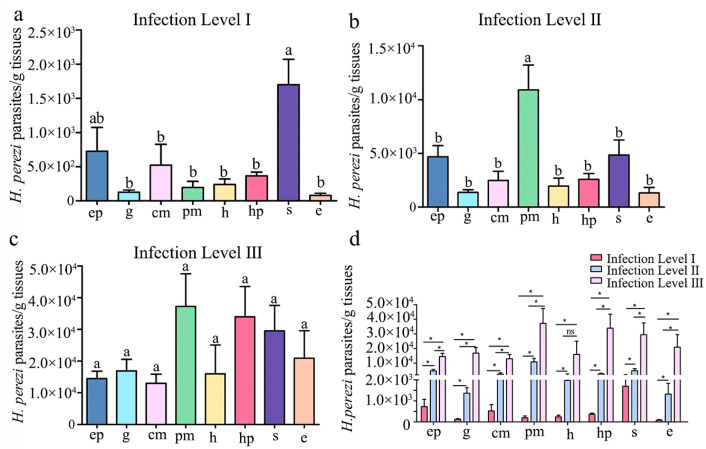
***H. perezi* abundance in eight tissues of *P. trituberculatus* with different infection levels.** *H. perezi* ITS1 copies detected in eight tissues of *P. trituberculatus* with infection levels I (**a**), II (**b**), and III (**c**) and a quantitative comparison of *Hematodinium* parasite load in eight tissues of *P. trituberculatus* with different infection levels based on qPCR results (**d**). Data are presented as the mean ± SD (*n* = 6). The bars in the same histogram without common lowercase letters on them indicate statistical significance (*p* < 0.05). ep, epidermis; g, gills; cm, cheliped muscle; pm, pereiopod muscle; h, heart; hp, hepatopancreas; s, stomach; e, eyestalks. *: *p* < 0.05, ns: *p* > 0.05.

The average abundance of *H. perezi* in tissues of the naturally infected *P. trituberculatus* with different infection levels was quantitatively analyzed by qPCR (Figure 4). In *P. trituberculatus* with infection level I, the average abundance of *H. perezi* was significantly higher in the stomach (1.7 × 10^3^ parasites/g; *p* < 0.05) than in other tissues including the epidermis (7.3 × 10^2^ parasites/g) and cheliped muscle (5.2 × 10^2^ parasites/g) (Figure 4a). In the individuals with infection level II, the highest abundance of *H. perezi* was detected in the pereiopod muscles (1.1 × 10^4^ parasites/g; *p* < 0.05), followed by the stomach (4.8 × 10^3^ parasites/g), epidermis (4.7 × 10^3^ parasites/g), and hepatopancreas (2.6 × 10^3^ parasites/g) (Figure 4b). In the individuals with infection level III, the highest abundance of *H. perezi* was found in the pereiopod muscles (3.5 × 10^4^ parasites/g), followed in a descending order by the hepatopancreas (3.4 × 10^4^ parasites/g), stomach (3.0 × 10^4^ parasites/g), eyestalks (2.1 × 10^4^ parasites/g), gills (1.7 × 10^4^ parasites/g), and heart (1.6 × 10^4^ parasites/g), while no significant difference was found in the abundance of *H. perezi* across the eight tissues (*p* > 0.05) (Figure 4c). Additionally, the qPCR results revealed significant differences in parasite load among the three infection levels (Figure 4d, Supplementary Table S3). In all eight examined tissues, *P. trituberculatus* classified as heavily infected (level III) exhibited markedly higher *Hematodinium* load compared to those with moderate (level II) and light (level I) infections.

## 4. Discussion

Parasitic dinoflagellates in the *Hematodinium* genus mainly live and proliferate in the hemolymph or hemocoel of major tissues in crustacean hosts, causing serious pathological changes in the organs/tissues of infected hosts [1,2,3,8]. Given the complex life history of *H. perezi*, elucidating the transition in its life stages and tissue tropism within crustacean hosts provides valuable insights into the in vivo interaction between *H. perezi* and crustacean hosts. In the present study, both qualitative and quantitative methods were employed to investigate the tissue distribution and parasite abundance of *Hematodinium perezi* in the hemolymph and eight organs of naturally infected *Portunus trituberculatus*. The life stages of *H. perezi* varied in the hemolymph of *P. trituberculatus* with different infection levels. Furthermore, there were differences in the tissue distribution patterns found in *P. trituberculatus* with different infection degrees, which suggested the tissue tropism of *H. perezi* in crustacean hosts and implied that hemolymph and stomach were likely two key colonization sites for *H. perezi* filamentous trophonts during the early infection period.

Filamentous trophonts were mainly detected in the hemolymph and stomach tissues in *P. trituberculatus* with infection level I in the present study. In previous studies, filamentous trophonts of *H. perezi* were found in the hemolymph as well as the tissues (e.g., heart, hepatopancreas) of lightly infected crustacean hosts such as snow crabs, shore crabs, and Chinese swimming crabs [9,24,28,33,34]. As indicated by the in vitro complete life history and transmission route of *Hematoidnium* parasites [25,26], the filamentous trophonts were developed and transformed from the dinospores which is the free-living life stage involved in invading susceptible crustacean hosts via waterborne transmission [35,36,37]. Previous studies have confirmed that dinospores can initiate infections, and sentinel experiments have further demonstrated successful transmission to blue crabs (*Callinectes sapidus*) and *Portunus trituberculatus* through exposure to infested water [37,38,39]. And our recent work has proved that the dinospores could invade and infect two susceptible crustacean hosts (*P. trituberculatus* and *Helice tientsinensis*) (unpublished data). Parasitism in the connective tissues surrounding the stomach may be advantageous for *H. perezi* to acquire nutrients from the crustacean hosts. Thus, we speculated that the hemolymph and the stomach tissues detected with *H. perezi* presence in the present study were likely important sites for *Hematodinium* colonization in crustacean hosts during the early infection period.

With progression to infection level II, the limited number of filamentous trophonts was replaced with a large number of round trophonts in the hemolymph and tissues of the naturally infected *P. trituberculatus* in the present study. The abundance of *H. perezi* was significantly much higher in the hemolymph of *P. trituberculatus* with infection level II than that of the individuals with infection level I. Amoeboid trophonts are a common and important in vivo proliferative life stage of *Hematodinium* parasites detected qualitatively in the hemolymph or multiple tissues (e.g., hepatopancreas, heart, gills, muscles) of crustacean hosts in previous studies [24,26,28]. In infected blue crabs, *Hematodinium* infection has also been associated with notable histological alterations in major organs such as the heart, gills, and hepatopancreas. Within these tissues, both uninucleate and multinucleate stages of *Hematodinium* have been identified, further suggesting active proliferation and tissue invasion [40]. So, it might be a crucial period for the fast proliferation of *H. perezi* in the hemolymph of *P. trituberculatus* at infection level II. In addition, the tissue with the highest *H. perezi* abundance in *P. trituberculatus* switched from that of the stomach to that of the pereiopod muscles with a dramatic 56-fold increase in the parasite number when infection level I developed to level II in the present study. In addition, the intramuscular adipose tissue could serve as an energy storage and metabolism repository in crustaceans [41], which might imply that pereiopod muscles were the preferred tissue site to potentially provide nutrients and energy for *H. perezi* at infection level II.

Compared to *H. perezi* in *P. trituberculatus* with infection level II, the life stage of *H. perezi* was transformed from trophonts to sporoblasts and proliferated in large amounts in all of the examined tissues of the individuals with infection level III, with a high parasite intensity of 3.1 × 10^7^ cells/mL in the hemolymph and a comparable, especially large parasite intensity of *H. perezi* sporoblasts (3.0–3.5 × 10^4^ cells/g) found in three tissues (pereiopod muscles, hepatopancreas, and stomach). As suggested by previous studies [41,42,43,44], these three tissues (pereiopod muscles, hepatopancreas, and stomach) and the hemolymph are importantly involved in the process of nutrient absorbtion, storage, and transportation in crustaceans, which likely account for the especially massive proliferation of *H. perezi* in these three tissues of *P. trituberculatus* with infection level III in this study. Then, the massive proliferation of *H. perezi* sporoblasts in *P. trituberculatus* with infection level III resulted in systematic infection with significant histopathological alterations in nearly all the tissues through the haemal sinuses and connective tissues [1,9,14]. The semi-closed circulatory system of crustaceans may have also contributed to the broad tissue distribution of *H. perezi* and the subsequent systemic histopathological alterations in the crabs with infection level II and III. The circulating hemolymph likely facilitated the efficient spread and infiltration of *H. perezi* in various tissues of the crustacean hosts. In addition, parasites employ mechanisms such as sequestration, extravasation, transcellular migration, and vascular permeability to migrate through different host tissues [45], while the mechanisms by which *Hematodinium* invades and disseminates within the host’s multiple tissues need further study.

Quantitative analysis results for *H. perezi* revealed a dramatic increase in the parasite abundance in the hemolymph of naturally infected *P. trituberculatus* during the infection period in this study. As a primary medium for nutrient distribution, hemolymph transports essential substances such as amino acids, sugars, and lipids throughout the hosts [46,47]. By residing in the hemolymph, *H. perezi* gained direct access to these resources to support its development. Furthermore, a primary function of crustacean hemocytes is to defend against disease agents and injury [46,47,48,49]. Like other invertebrates, crustaceans primarily depend on innate immunity to combat foreign invasive pathogens [48,49]. And crustacean hemocytes are involved in series of host immune responses including recognition, phagocytosis, melanization, cytotoxicity, and cell–cell communication, playing central roles in hosts’ immunity against foreign substances and organisms [50,51]. In addition, *Hematodinium* infections are consistently associated with reduced hemocyte counts across various marine crustaceans [52,53], and the reduction in crustacean hemocytes caused by *Hematodinium* infection impaired the normal host immunity and renders it susceptible to secondary infections [15]. In the present study, a progressive and severe reduction in hemocytes was observed in the hemolymph of the *P. trituberculatus* with *Hematodinium* infection level II and III, which was consistent with previous studies [15,54]. The significant reduction in the hemocytes in the present study likely facilitated the tissue proliferation of *H. perezi* in *P. trituberculatus* with infection level II and III.

In addition, there existed some mismatch between histology and qPCR assays of *H. perezi* presence in nearly all tissues except for in the stomach of *P. trituberculatus* with infection level I and the gills of individuals with infection level II in this study. During the early infection level I, the mismatch in the results of *H. perezi* presence between the two detection methods was mainly attributed to the difference in the detection sensitivity of the two techniques themselves. The histology mainly provided direct and qualitative microscopic observations of *Hematodinium* presence in tissues containing a certain amount of parasite cells, while the molecular qPCR assay had much higher sensitivity and lower detection limitation (0.3 parasites/100 μL hemolymph) [30] and the advantage of quantifying the *Hematodinium* presence. The qPCR assay demonstrated higher sensitivity compared to histological analysis, enabling the detection and quantification of *Hematodinium* parasites even in early and low-level infections. This molecular approach provided important complementary information that is often missed by conventional histology. Meanwhile, the absence of *H. perezi* in the gills of *P. trituberculatus* with infection level II according to histology analysis in this study was likely due to the function and nutritional condition of this tissue besides the detection sensitivity of techniques. The gills predominantly serve as the respiratory organ to obtain oxygen via water exchange and are not nutrient-rich. Thus, the frequent water exchange and the less favorable nutrient conditions in the gills possibly hampered the colonization of *H. perezi* in the gills of *P. trituberculatus* with infection level II. When *H. perezi* proliferated to a massive extent in crustacean hosts, *H. perezi* presence could be observed in the gills via the infiltration of this parasite, as shown in the present study.

Meanwhile, the in vitro life stages of *Hematodinium* parasites [20,24,25,26] was not exactly the same as the in vivo findings in their crustacean hosts including Norway lobsters, blue crabs, Chinese swimming crabs, and mudflat crabs [7,25,26,28], with some in vitro life stages (e.g., Arachnoid sporonts, Schizonts, and Gorgonlocks colony) not found in crustacean hosts. The differences in the development of the *Hematodinium* life cycle might be mainly due to the distinct survival microenvironment for *Hematodinium* parasites. The nutrient components of the in vitro culture medium were not exactly the same as those in the crustacean hosts, and the supplementary components (e.g., saline buffer, antibiotics), the initial cultured cell density, and the culture duration could potentially influence the development of *Hematodinium* parasites. Most importantly, the in vitro culture was totally detached from the complex in vivo interaction and exchange process (e.g., immune evasion, metabolic exchange) between *Hematodinium* and its crustacean hosts with different infection status. Thus, the life history cycle depicted by in vitro observation could not completely reflect that in crustacean hosts under real infections. And we propose to collect a larger sample of crustacean hosts with more *Hematodinium* infection stages to systematically investigate the in vivo life history of *H. perezi* in future studies.

In conclusion, the present study investigated the life stages, tissue distribution, and parasite abundance of naturally *H. perezi*-infected *P. trituberculatus*. Filamentous trophonts of *H. perezi* were mainly observed in the hemolymph and stomach of crabs with infection level I, which suggested that the two tissues were likely important colonization sites for *H. perezi* during the early infection stage. In addition, *H. perezi* exhibited tissue-specific variation in infection intensity across the hemolymph and eight organs of naturally infected *P. trituberculatus*. *H. perezi* trophonts and sporoblasts predominated in the hemolymph of the crabs with infection levels II and III, respectively. The top three tissues with high *H. perezi* abundance were those of the pereiopod muscles, stomach, and epidermis in crabs with infection level II, while these were those of the pereiopod muscles, hepatopancreas, and stomach in the individuals with infection level III, which suggested the tissue tropism of *H. perezi* in crustacean hosts during its infection period. Furthermore, the infection classification used in this study was based solely on parasite abundance in hemolymph smears. While practical, this approach remains limited in scope. Future work should aim to establish a more comprehensive and standardized infection grading scale that incorporates multiple histological, molecular, and quantitative criteria, which would greatly benefit future research on *Hematodinium* pathogenesis and diagnostics.

## Data Availability

The original contributions presented in this study are included in the article/Supplementary Materials. Further inquiries can be directed to the corresponding author.

## Supplementary Materials

The following supporting information can be downloaded at: https://www.mdpi.com/article/10.3390/pathogens14070650/s1, Figure S1: The standard curve for the qRT-PCR assay of *H. perezi* abundance in this study; Table S1: Comparison of the developmental stages of *Hematodinium perezi* observed in this study and the other several previous studies; Table S2: Classification criteria of *Hematodinium perezi* infection levels in *P. trituberculatus* based on microscopic examination (200× field) as described in [20]; Table S3: Comparison of *H. perezi* abundance in various tissues across different infection levels by qPCR analysis. Statistical significance was considered as *p* < 0.05.
