# Supplementary material for: Tissue Distribution and Abundance of the Parasitic Dinoflagellate Hematodinium perezi in Naturally Infected Portunus trituberculatus
Source: Pathogens. 2025 Jun 30;14(7):650. doi: 10.3390/pathogens14070650 (PMC12301046; doi:10.3390/pathogens14070650)
Supplement: Supplementary file 1 [file pathogens-14-00650-s001.zip › pathogens-3671077-supplementary.pdf]

Supplementary Figure S1

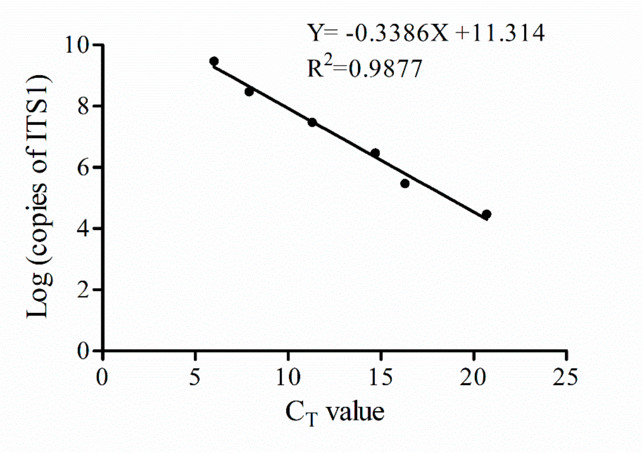

Figure S1. The standard curve for the qRT-PCR assay of *H. perezi* abundance in this study. The measurement was conducted in triplicate for each sample. The regression equation was Y = -0.3386X + 11.314 (R^2 = 0.9877).

Supplementary Table S1

Table S1. Comparison of the developmental stages of *Hematodinium perezi* observed in this study and the other several previous studies. Y: observed, N: Not observed.

Life Stages	Observed *in vivo* (This study)	Observed *in vivo* (Previous studies)	Observed *in vitro* (Previous studies)	References
Filamentous trophonts	Y	Y	Y	[20, 22, 24]
Amoeboid trophonts	Y	Y	Y	[20, 22, 24]
Arachnoid trophonts	N	Y	Y	[20, 22, 24]
Schizonts	N	N	Y	[20]
Clump colonies	N	Y	Y	[20, 22, 24]
Arachnoid sporonts	N	N	Y	[20]
Sporoblasts	Y	Y	Y	[20]
Prespores	N	Y	Y	[22]
Dinospores	N	Y	Y	[24]

Supplementary Table S2

Table S2. Classification criteria of *Hematodinium perezi* infection levels in *P. trituberculatus* based on microscopic examination (200× field) as described in Wang et al. 2017 [20].

Infection Level	Parasite count under 200× magnification field	Parasite count under 200× magnification field (this study）
Level I	< 10	4 ± 2
Level II	10 -100	80 ± 10
Level II	> 100	200 ± 35

Supplementary Table S3

Table S3. Comparison of *H. perezi* abundance in various tissues across different infection levels by qPCR analysis. Statistical significance was considered as $p < 0.05$.

Tissues	*p*-value (level I vs II）	*p*-value (level I vs III）	*p*-value (level II vs III）
Epidermis	0.011	0.013	0.018
Gills	0.005	0.022	0.025
Cheliped muscle	0.04	0.025	0.030
Pereiopod muscle	0.005	0.035	0.049
Heart	0.038	0.046	0.132
Hepatopancreas	0.007	0.036	0.041
Stomach	0.048	0.037	0.044
Eyestalks	0.035	0.036	0.043
